# Supplementary material for: Effect of fermentation stages on glucosinolate profiles in kimchi: Quantification of 14 intact glucosinolates using ultra-performance liquid chromatography-tandem mass spectrometry
Source: Food Chem X. 2022 Aug 10;15:100417. doi: 10.1016/j.fochx.2022.100417 (PMC9532793; doi:10.1016/j.fochx.2022.100417)
Supplement: Supplementary data 1 [file mmc1.docx]

**Research Article**

**Effect of fermentation stages on glucosinolate profiles in kimchi: Quantification of 14 intact glucosinolates using ultra-performance liquid chromatography-tandem mass spectrometry**

Su-Yeon KIM ^a^, Jisu YANG ^b^, Yun-Mi DANG ^a^, Ji-Hyuong HA ^a,^*

^a^ Hygienic Safety and Distribution Research Group, World Institute of Kimchi, Gwangju 61755, Republic of Korea

^b^ Industrial Solution Research Group, World Institute of Kimchi, Gwangju 61755, Republic of Korea

*Corresponding author. Tel: +82 62 610 1845; E-mail address: hajee@wikim.re.kr

Email addresses: sykim17@wikim.re.kr (S. Y. KIM), yangjs@wikim.re.kr (J. YANG), ymdang@wikim.re.kr (Y. M. DANG), hajee@wikim.re.kr (J. H. Ha)

**Abstract**

We developed and validated an ultra-performance liquid chromatography-electrospray ionization tandem mass spectrometry-based analytical method to determine intact glucosinolates in kimchi and evaluate the effects of fermentation stages on glucosinolate profiles. The developed method yielded reliable data in the kimchi matrix in terms of selectivity, matrix effect (88%–105%), linearity (coefficients of determination ≥0.9991), sensitivity (limits of quantification ≤35 nmol/L), accuracy (82%–101%), and precision (≤8%). The kimchi samples contained progoitrin, sinigrin, glucoraphanin, glucoraphenin, glucoalyssin, gluconapin, glucobrassicanapin, glucobrassicin, glucoberteroin, gluconasturtiin, 4-methoxyglucobrassicin, and neoglucobrassicin, of which 4-methoxyglucobrassicin, glucobrassicanapin, and gluconapin were the major compounds. Total glucosinolate content was decreased by 31%–97% and 91–100% in the moderate-fermented and over-fermented samples, respectively, compared with that in the non-fermented samples, revealing sudden glucosinolate degradation between the moderate- and over-fermentation stages. In summary, we report an efficient analytical method to estimate kimchi glucosinolate profiles, which could be a foundation for future studies.

***Keywords:*** cruciferous vegetables, electrospray ionization tandem mass spectrometry, fermented foods, glucosinolate degradation, kimchi cabbage, method validation

**1. Introduction**

Regular consumption of cruciferous vegetables has been proposed to benefit human health, including cancer prevention and anti-inflammatory effects, mainly attributed to glucosinolate-derived isothiocyanates and indoles (Fuentes et al., 2015; Esteve, 2020). Glucosinolates are sulfur-containing secondary metabolites abundant in cruciferous vegetables, such as broccoli, cabbage, and other green leafy vegetables and are responsible for their characteristic smell and taste. These compounds are biologically inert; however, they can be degraded into isothiocyanates, nitriles, epithionitriles, thiocyanates, and indoles, based on the type of glucosinolate, environmental pH, and the presence of specific proteins. The degradation is caused by coexisting myrosinase, a thioglucosidase, activated upon maceration of tissues (Hanschen et al., 2014). Intact glucosinolates can also partly be metabolized to form breakdown products such as isothiocyanates by the myrosinase-like activity of the human gut microbiota (Shakour et al., 2022).

Kimchi, listed in the Codex Alimentarius in 2001 (CODEX STAN 223-2001), is a globally known traditional fermented food. Kimchi constitutes a major component of Korean food and is composed of fermented vegetables, primarily brined cabbage (*Brassica rapa* L. subsp. *pekinensis*) and mixed with various seasonings such as red pepper (*Capsicum annuum* L.) powder, garlic, ginger, and edible Allium varieties. In Korea, kimchi is frequently consumed in substantial amounts (the average daily intake of kimchi was approximately 64 g in 2015–2019) [National Nutrition Examination Survey 2015–2019, Korea Health Industry Development Institute (KHIDI)]. Therefore, it is likely to be the primary dietary source of glucosinolates and their breakdown products in Korea. Moreover, Korean kimchi exports have increased drastically in recent years (Korea agricultural trade information (KATI), 2020), indicating its increased global consumption and popularity. Therefore, to realize the therapeutic potentials of kimchi, investigation and estimation of the glucosinolates and their breakdown products in kimchi is important.

Total glucosinolate content in kimchi cabbage varies in the range of 2.70–57.88 µmol/g dry weight (DW) based on the variety, and gluconapin, glucobrassicanapin, and 4-methoxyglucobrassicin are the major glucosinolates (Baek et al., 2016; Kim et al., 2010; Lee et al., 2014; Chun et al., 2018). Glucosinolates in kimchi cabbage can be partly lost because of their interaction with myrosinase or leaching during kimchi preparation, which involves trimming, cutting, salting, and seasoning, thus damaging plant tissues. Moreover, during kimchi fermentation, glucosinolates can be further degraded into breakdown products by myrosinase and myrosinase-like bacterial enzymes (Hanschen et al., 2014). Collectively, various factors, including processing and fermentation conditions and the intrinsic quality of kimchi cabbage, which is determined by the variety, growth, and storage conditions, are the major determinants of the glucosinolate content of kimchi. However, the glucosinolate profile of kimchi and the factors affecting it have garnered less attention. To date, only one study has reported relative quantities of glucosinolates in kimchi products (Kim et al., 2017). This study identified glucoalyssin (0.00–7.07 µmol/g DW), gluconapin (0.00–5.85 µmol/g DW), glucobrassicanapin (0.00–11.87 µmol/g DW), glucobrassicin (0.00–0.42 µmol/g DW), and 4-methoxyglucobrassicin (0.12–9.36 µmol/g DW) in kimchi samples; however, it did not consider the effects of various fermentation processes on the glucosinolate contents.

Glucosinolates are typically determined by analyzing desulfoglucosinolates after the enzymatic desulfation of intact glucosinolates using reversed-phase liquid chromatography coupled with ultraviolet or diode array detection (Hennig et al., 2012; Klopsch et al., 2017). However, with the advancement of technologies, electrospray ionization mass spectrometry (ESI-MS), which avoids the time-consuming and poorly controlled desulfation step (Bernal et al., 2019; Hooshmand & Fomsgaard, 2021), has been used for the profiling of intact glucosinolates. Moreover, ultra-performance liquid chromatography (UPLC) and ultra-high-performance liquid chromatography have been shown to improve the resolution and sensitivity of the techniques and enable faster separation of glucosinolates (Thomas et al., 2018; Capriotti et al., 2018; Bernal et al., 2019). The complexity of the kimchi matrix comprising various ingredients (Codex Alimentarius Commission, 2001), makes it difficult to obtain reliable and accurate results. Therefore, determining glucosinolates in kimchi requires an efficient clean-up process to eliminate substances that interfere with this analysis.

We hypothesized that developing and validating an efficient analytical method using UPLC-ESI-tandem mass spectrometry (MS/MS) can efficiently determine intact glucosinolates in kimchi. To test this hypothesis, we aimed to develop and validate a method based on UPLC-ESI-MS/MS and assess the effects of fermentation stages on the glucosinolate profiles of kimchi. To the best of our knowledge, this study is the first to determine the effect of fermentation stage on glucosinolate profiles in kimchi using a validated analytical method for quantification of glucosinolates in intact forms. This study is expected to contribute to our understanding of changes in glucosinolates during kimchi fermentation and improve the efficiency of glucosinolate quantification in kimchi.

**2. Materials and methods**

*2.1. Reagents and chemicals*

HPLC-grade methanol and acetonitrile were purchased from J. T. Baker. Extra-pure ammonia solution (≥28.0%) was purchased from Junsei (Saitama, Japan). ACS-reagent-grade formic acid (≥98%) and sinigrin hydrate (≥99.0%) were purchased from Sigma-Aldrich. Other glucosinolates, including glucoiberin potassium salt (≥99.0%), glucocheirolin potassium salt (≥97.0%), progoitrin potassium salt (≥97.0%), glucoraphanin potassium salt (≥97.0%), glucoraphenin potassium salt (≥97.0%), glucoalyssin potassium salt (≥97.0%), gluconapin potassium salt (≥97.0%), glucobrassicanapin potassium salt (≥98.0%), glucobrassicin potassium salt (≥97.0%), glucoberteroin potassium salt (≥97.0%), gluconasturtiin potassium salt (≥97.0%), 4-methoxyglucobrassicin potassium salt (≥94.0%), and neoglucobrassicin potassium salt (≥97.0%) were purchased from PhytoPlan^®^ (Heidelberg, Germany). The structures of the glucosinolates are shown in Figure S1. Glucosinolate standard solutions for calibration were prepared using deionized water, and their concentrations were as follows: 7, 10, 70, 300, 700, and 1,000 nmol/L for sinigrin, gluconapin, glucobrassicanapin, progoitrin, glucoiberin, gluconasturtiin, glucoberteroin, glucoraphanin, glucocheirolin, glucobrassicin, and glucoalyssin; 10, 70, 300, 700, and 1,000 nmol/L for glucoraphenin; 7, 10, 70, 300, 700, 1000, and 3000 nmol/L for 4-methoxyglucobrassicin; and 3, 5, 7, 10, 70, 300, 700, and 1,000 nmol/L for neoglucobrassicin.

*2.2. Sample preparation*

Twenty kimchi products freshly prepared from kimchi cabbage were purchased from various manufacturers in Korea. Titratable acidities (as lactic acid) of the filtrates of homogenized kimchi samples (10 mL) were measured by titrating with 0.1 N sodium hydroxide solution to pH 8.3 using an automatic titrator (Model TitroLine 5000; SI Analytics, Mainz, Germany). These kimchi samples had titratable acidity of <0.5% and were classified as non-fermented kimchi. To obtain moderate-fermented and over-fermented kimchi, the collected kimchi samples were stored at 6 °C until titratable acidities were either ≥0.6 and ≤1.0 (moderate-fermented kimchi), which took 1−2 weeks; or >1.0% (over-fermented kimchi), which took more than 3 weeks. The fermentation stages were based on those proposed by Korea Food Research Institute (KFRI, 2006).

*2.3. Sample treatment*

Approximately 50 mg of each freeze-dried and ground sample was transferred into a 15 mL conical tube with a cap and mixed with 10 mL 70% (v/v) methanol. The mixture was extracted by sonication for 10 min at room temperature and then centrifuged at 3,900 rpm for 20 min at 4 °C. After removing methanol from the collected supernatant using a rotary evaporator, the volume of the remaining aqueous phase was adjusted to 10 mL with deionized water. The resulting solution was filtered through a 0.20 μm syringe filter. For clean-up, 2 mL of the filtrate was loaded onto a weak anion-exchange solid-phase extraction (SPE) cartridge (Oasis WAX 3 cc cartridge, weight: 60 mg, particle size: 30 µm, Waters) that was previously conditioned with 3 mL methanol and activated with 3 mL 2% (v/v) formic acid. The cartridge was sequentially washed with 1 mL 2% formic acid and 1 mL methanol. After drying for 2 min under vacuum, the analytes were eluted with 5% (v/v) ammonia solution (≥28.0%) in methanol (10 mL). The eluted solution was thoroughly dried by rotary evaporation and reconstituted with 2 mL deionized water. The resulting solution was used for UPLC-ESI-MS/MS analysis.

*2.4. UPLC-ESI-MS/MS analysis*

Intact glucosinolates were quantified using an Acquity UPLC^®^ I-Class system coupled with a tandem quadrupole mass spectrometer (Xevo TQ-S) equipped with an ESI source. Chromatographic separation was performed using an Acquity UPLC^®^ BEH C18 column (2.1 × 150 mm, 1.7 µm, Waters) with a mixture of 0.1% (v/v) formic acid in water (A) and 0.1% (v/v) formic acid in acetonitrile (B) as the mobile phase using the following linear gradient conditions: 100% (v/v) of A for 0–1 min, 100% to 95% of A for 1–3 min, 95% to 70% of A for 3–6.2 min, 70% of A for 6.2–7.2 min, 70% to 0% of A for 7.2–8 min, 0% of A for 8–9 min, 0% to 100% of A for 9–10 min, and 100% of A for 10–12 min. The column temperature was maintained at 30 °C, the flow rate was 0.25 mL/min, and the injection volume was 1 µL. ESI was performed with the negative-ion mode under the following conditions: capillary voltage, 3 kV; desolvation temperature, 350 °C; desolvation gas flow, 650 L/h; cone gas flow, 150 L/h; source temperature, 150 °C.

*2.5. Method validation*

Method validation was performed according to international guidelines (Association of Analytical Communities (AOAC), 2012; United States Food and Drug Administration (USFDA), 2019). To determine the selectivity of the proposed method, the chromatograms of the samples were compared with those of standard solutions and samples spiked with standard solutions. The limit of detection (LOD) and quantification (LOQ) values were calculated using the following formula:

LOD = 3.3[σ/S],

LOQ = 10 [σ/S],

where σ and S are the standard deviation of the y-intercept and the slope obtained from triplicate calibration curves with 5–8 points for each analyte, respectively. The calibration ranges were as follows: 7–1,000 nmol/L for sinigrin, gluconapin, glucobrassicanapin, progoitrin, glucoiberin, gluconasturtiin, glucoberteroin, glucoraphanin, glucocheirolin, glucobrassicin, and glucoalyssin; 10–1,000 nmol/L for glucoraphenin; 7–3000 nmol/L for 4-methoxyglucobrassicin; and 3–1,000 nmol/L for neoglucobrassicin.

The matrix effect was evaluated by comparing the analyte concentrations in the blank matrix (sample dissolution solvent; deionized water) spiked with standard solutions at three concentration levels (low, 100 nmol/L; medium, 300 nmol/L; and high, 500 nmol/L) to those in the sample matrix spiked with standard solutions of the same concentrations after extraction. The matrix effect (n = 6) was calculated using the following formula:

Matrix effect (%) = [(*A* – *B)*/*C]* × 100,

where *A* is the analyte concentration in the sample matrix spiked after extraction, *B* is the analyte concentration in the non-spiked sample matrix, and *C* is the analyte concentration in the spiked blank matrix (sample dissolution solvent; deionized water).

The accuracy and the intraday and interday precision were evaluated by comparing the analyte concentrations in the samples spiked with standard solutions of three concentrations (low, 100 nmol/L; medium, 300 nmol/L; and high, 500 nmol/L) after extraction to those in the samples spiked with standard solutions of the same concentrations before extraction. The accuracy and intraday precision data were collected on the same day (n = 6), whereas the interday precision data were collected on three consecutive days (n = 6). The accuracy was expressed as a percentage of recovery using the following formula:

Recovery (%) = [(*A* − *C*) / (*B* − *C*)] × 100,

where *A* is the analyte concentration in the sample matrix spiked before extraction, *B* is the analyte concentration in the sample matrix spiked after extraction, and *C* is the analyte concentration in the non-spiked sample matrix. Precision was expressed as relative standard deviation (RSD) values.

*2.6. Statistical analysis*

Means and standard deviations of data were calculated using IBM SPSS Version 19.

**3. Results and discussion**

*3.1. UPLC*

Several experiments were performed to establish efficient gradient elution conditions using a mixture of standard solutions, different mobile phase compositions, and flow rate conditions. The best results were obtained using the mobile phase gradient described in section 2.4. As shown in Figure S2, the chromatographic separation of 14 intact glucosinolates was completed within 9 min. The overall run time required to obtain a reproducible retention time was 12 min, which is shorter than that reported previously (Thomas et al., 2018; Bernal et al., 2019; Hooshmand & Fomsgaard, 2021).

*3.2. ESI-MS/MS*

Optimal multiple reaction monitoring parameters for the 14 intact glucosinolates were established and are summarized in Table S1. The optimal operating parameters (i.e., cone voltage and collision energy) for the two most intense transitions (one precursor ion → two product ions) for each intact glucosinolate were determined by directly injecting each standard solution into the mass spectrometer operated in negative ionization mode. The most abundant product ion formed from each intense precursor [M-H]^−^ occurred at *m*/*z* 97, corresponding to the sulfate moiety of glucosinolates, and was selected to quantify the intact glucosinolates. The second most abundant product ion was monitored together with the product ion at *m*/*z* 97 to identify intact glucosinolates. The ions for confirmation of analytes are summarized in Table S1. The precursor and product ions selected in this study for intact glucosinolate analysis are commonly used in an MS/MS system (Thomas et al., 2018; Capriotti et al., 2018).

*3.3. Sample extraction and clean-up treatment*

Intact glucosinolates were extracted by the ultrasonic extraction method using 70% (v/v) methanol as the extraction solvent. To determine the appropriate extraction time, the extractions were performed for 10, 30, and 60 min. The optimal extraction time was found to be 10 min, as no remarkable improvements in the extraction efficiency were observed when longer extraction times were used.

A weak anion-exchange SPE cartridge was used for the extraction clean-up process. Several solvents were evaluated to establish an effective SPE procedure. For the washing step, combinations of 1 or 2 mL 2% (v/v) formic acid in water followed by 0.5 or 1 mL methanol were tested. The most effective solvents were 1 mL 2% formic acid in water, followed by 1 mL methanol. For the elution step, suitable recoveries (80%–110%) were obtained using 10 mL 5% (v/v) ammonia solution (≥28.0%) in methanol. The established SPE treatment was effective in eliminating interference that affected the matrix effect (see section 3.4.2).

*3.4. Validation of the proposed method*

3.4.1. Selectivity

To evaluate the selectivity of the proposed method, the chromatograms of the standard solutions were compared with those of kimchi samples spiked with a mixture of standard solutions, as obtaining a glucosinolate-free kimchi sample as a blank matrix was impossible (Figure S2). No interfering peaks were observed at the retention time for any intact glucosinolates, indicating the absence of interference from coexisting matrix components. These results indicated that the proposed method is selective for determining intact glucosinolates in kimchi.

3.4.2. Matrix effect

To evaluate the effect of the kimchi matrix on the ESI process, we assessed the matrix effect in the kimchi extract, and significant ion suppression or enhancement was observed for several intact glucosinolates when the clean-up treatment was not performed (Table S2). These results indicated that co-eluting compounds that change the ionization efficiencies and affect the quantification of intact glucosinolates might be present in the kimchi matrix. In addition, sample clean-up is required to eliminate interference, even when using MS/MS, one of the most sensitive and selective detection systems, because of the unavoidability of accompanying matrix effects during the analysis of complicated matrices using ESI (Matuszewski et al., 2003; Zhou et al., 2017). Therefore, we attempted to improve the matrix effect using SPE treatment. This strategy seemed more suitable than reducing the injection volume, diluting the sample, or using matrix-matched calibration. Furthermore, the concentrations of some glucosinolates in the kimchi matrix might not be high enough to facilitate quantification after dilution. Moreover, the relative expense of the standard solutions makes the matrix-matched calibration expensive for the analysis of large numbers of samples.

The use of SPE treatment successfully eliminated unintended interference in the kimchi matrix. It improved the matrix effects to 98%–105% for the intact glucosinolates at the three spiked concentrations, except for that with 4-methoxyglucobrassicin, for which the matrix effect was 88%–91% (Table 1). These results confirmed the absence of interference that might considerably alter the ESI process, indicating that the proposed method is selective for determining intact glucosinolates in the kimchi matrix.

3.4.3. Linearity and sensitivity

The calibration curves of all intact glucosinolates showed excellent coefficients of determination (r^2^ ≥ 0.9991; Table 2). Moreover, the LOD and LOQ values of all intact glucosinolates were lower than 11 and 35 nmol/L, respectively, which were lower than those reported previously (Bernal et al., 2019; Hooshmand & Fomsgaard, 2021; Maldini et al., 2017).

3.4.4. Accuracy

Table 3 summarizes the accuracy results, as evaluated for the recovery of intact glucosinolates from the kimchi matrix. The recovery of intact glucosinolates was in the range of 83%–92% at the low concentration, 82%–95% at the medium concentration, and 86%–101% at the high concentration. The recovery of all intact glucosinolates was within the range of 80%–110%, which is considered acceptable according to international guidelines (AOAC International, 2012; USFDA, 2019).

3.4.5. Intraday and interday precision

The intraday and interday RSD values for the 14 intact glucosinolates at the three spiked concentrations were used to assess the precision of the proposed method (Table 4). The intraday RSD values ranged from 3% to 8% at the low concentration, 3% to 8% at the medium concentration, and 2% to 7% at the high concentration. The interday RSD values were in the range of 5%–8% at the low concentration, 4%–8% at the medium concentration level, and 2%–7% at the high concentration. All these values were ≤8% and satisfied the requirements of international guidelines (AOAC International, 2012; USFDA, 2019).

*3.5. Concentrations of intact glucosinolates in kimchi with different fermentation stages*

Table 5 summarizes the results of intact glucosinolate profiling in kimchi samples with different fermentation stages performed based on the proposed analytical method. Twelve glucosinolates, namely progoitrin, sinigrin, glucoraphanin, glucoraphenin, glucoalyssin, gluconapin, glucobrassicanapin, glucobrassicin, glucoberteroin, gluconasturtiin, 4-methoxyglucobrassicin, and neoglucobrassicin, were identified in the kimchi samples, whereas glucoiberin and glucocheirolin were not detected in any of the samples. In non-fermented kimchi samples, the major compounds were 4-methoxyglucobrassicin (219 ± 3–1483 ± 36 nmol/g DW), glucobrassicanapin (37 ± 1–1318 ± 3 nmol/g DW), and gluconapin (5 ± 0–906 ± 6 nmol/g DW), and the average of their proportions accounted for 72.9% of the total glucosinolate content (Figure 1). These three compounds have been previously reported as the predominant glucosinolates in kimchi cabbage (Baek et al., 2016; Kim et al., 2010; Lee et al., 2014; Chun et al., 2018). The presence of 4-methoxyglucobrassicin as a predominant compound in kimchi throughout the fermentation process was remarkable because this compound is not known to be a major glucosinolate in other cruciferous vegetables (Agerbirk et al., 2009). Other glucosinolates such as neoglucobrassicin, glucoalyssin, glucobrassicin, progoitrin, glucoberteroin, and gluconasturtiin accounted for an average of 26% of the total glucosinolate content (Figure 1). Minor glucosinolates, such as glucoraphanin and glucoraphenin varied from <LOD to 11±0 nmol/g DW and ND to 81 ± 1 nmol/g DW, respectively (Table 5) and cumulatively accounted for 1% of the total glucosinolate content (Figure 1). Sinigrin was detected at non-quantifiable levels in most samples (Table 5).

Total glucosinolate contents of non-fermented samples varied from 443 ± 5 to 3606 ± 15 nmol/g DW, which is relatively lower than those of kimchi products reported (0.12–34.56 µmol/g DW; Kim et al., 2017) and kimchi cabbage (2.70–57.88 µmol/g DW; Baek et al., 2016; Kim et al., 2010; Lee et al., 2014; Chun et al., 2018). This difference could be attributed to various factors such as the inherent properties of kimchi cabbage, other kimchi ingredients, and kimchi manufacturing and fermentation processes. Total glucosinolate content decreased by 31%–97% in moderate-fermented samples (27 ± 1–1988 ± 8 nmol/g DW) and 91%–100% in over-fermented samples (0 ± 0–306 ± 8 nmol/g DW) compared with that in non-fermented samples.

Furthermore, the degradation rates of individual glucosinolates varied from 1% to 100% in moderate-fermented samples and 83% to 100% in over-fermented samples, where most glucosinolates were present at non-quantifiable levels. Moderate-fermented samples showed proportions of individual glucosinolates similar to those of non-fermented samples, whereas over-fermented samples showed proportions considerably different from those of non-fermented samples. Minor glucosinolates in kimchi samples, such as glucoraphanin and glucoraphenin, were completely degraded between non- and moderate-fermentation stages, probably because of their small amounts. In contrast, other glucosinolates such as progoitrin, gluconapin, glucobrassicin, glucoberteroin, gluconasturtiin, and neoglucobrassicin were nearly decomposed between moderate- and over-fermentation stages. However, 4-methoxyglucobrassicin, one of the major compounds, was detected in small quantities in most over-fermented samples (Table 5). These results suggested that sudden degradation of glucosinolates might have occurred between the moderate- and over-fermentation stages rather than between the non- and moderate-fermentation stages. However, rapid degradation of glucosinolates was observed in small amounts in the earlier fermentation stage. Drastic decomposition observed between moderate- and over-fermentation stages might be because of the release of glucosinolates through the gradual softening of fibrous structures of kimchi cabbage during fermentation (Ciska et al., 2021) and increased activities of lactic acid bacteria capable of metabolizing glucosinolates at a later stage of fermentation (Mullaney et al., 2013).

The difference in degradation rates led to a considerable difference in the general glucosinolate profile of kimchi samples. Because of these differences, the average proportion of the most dominant compound, 4-methoxyglucobrassicin, to the total glucosinolate content increased from 38.2% to 47.2% and then to 81.7%, as fermentation progressed (Figure 1). The relatively high stability of 4-methoxyglucobrassicin has also been reported in previous studies (Ciska et al., 2021; Tolonen et al., 2002). Considering that the degradation rate of individual glucosinolates can vary depending on factors such as chemical and microbiological stabilities of the respective compounds (Ciska et al., 2021), the presence of 4-methoxyglucobrassicin in most of the over-fermented samples might be related to the intrinsic abundance of the compound and/or perhaps the relatively high stability of kimchi fermentation under acidic conditions (Ciska et al., 2021; Tolonen et al., 2002).

Indole glucosinolates such as glucobrassicin, 4-methoxyglucobrassicin, and neoglucobrassicin can be metabolized into indole-3-carbinol, 4-methoxyindole-3-carbinol, and 1-methoxyindole-3-carbinol, respectively, and their potential health benefits such as anti-inflammatory and anti-cancer activities have been reported; however, most studies on the effects of indoles have exclusively focused on indole-3-carbinol (Fuentes et al., 2015; Agerbirk et al., 2009; Kronbak et al., 2010). In the present study, the proportion of these indole glucosinolates accounted for 49.5% in the non-fermented samples, 53.5% in the moderate-fermented samples, and 87.5% in the over-fermented samples. Among bioactive isothiocyanates, 4-methylsulfinylbutyl isothiocyanate (sulforaphane) and phenethyl isothiocyanate, the derivatives of glucoraphanin and gluconasturtiin, respectively, are the best-known compounds (Fuentes et al., 2015). However, in our samples, these two precursor compounds were found in small proportions. Various glucosinolates such as sinigrin, progoitrin, gluconapin, glucobrassicanapin, glucobrassicin, and neoglucobrassicin are known to be related to the characteristic bitterness and pungency of cruciferous vegetables, although these perceptions are not universal, whereas glucoraphanin lacks bitterness (Bell et al., 2018; Padilla et al., 2007). Because of the abundance of precursor glucosinolates, some breakdown products, including indoles such as 4-methoxyindole-3-carbinol from 4-methoxyglucobrassicin; 4-pentenyl isothiocyanate, 5-hexenenitrile, and 5,6-epithiohexanenitrile from glucobrassicanapin; and 3-butenyl isothiocyanate, 4-pentenenitrile, and 4,5-epithio-pentanenitrile from gluconapin might partly contribute to the characteristics of kimchi.

**4. Conclusions**

In the present study, we successfully developed and validated an analytical method based on UPLC-ESI-MS/MS. The developed method reliably identified and quantified the intact glucosinolates in kimchi. In addition, the general glucosinolate profiles of kimchi drastically changed after the moderate-fermentation stage, and several glucosinolates were present until the over-fermentation stage. However, for further understanding of the glucosinolates of kimchi, studies on various factors such as the intrinsic properties, storage, processing, fermentation, and distribution conditions of kimchi cabbage are needed. The proposed method would facilitate efficient and reliable evaluation of kimchi glucosinolates, and our results can provide data for further research.

**Declaration of competing interest**

The authors declare that they have no known competing financial interests or personal relationships that could have appeared to influence the work reported in this paper.

**Acknowledgments**

This work was supported by grants from the World Institute of Kimchi (KE2102-2 and KE2202-2), funded by the World Institute of Kimchi funded by the Ministry of Science and ICT, Republic of Korea.

**References**

Agerbirk, N., De Vos, M., Kim, J. H., & Jander, G. (2009). Indole glucosinolate breakdown and its biological effects. *Phytochemistry Reviews, 8*(1), 101–120. doi:[10.1007/s11101-008-9098-0](https://doi.org/10.1007/s11101-008-9098-0)

AOAC International. (2012). AOAC official methods of analysis. Guidelines for dietary supplements and botanicals. Retrieved March 25, 2022. <http://www.eoma.aoac.org/app_k.pdf>

Baek, S. A., Jung, Y. H., Lim, S. H., Park, S. U., & Kim, J. K. (2016). Metabolic profiling in Chinese cabbage (*Brassica rapa* L. subsp. pekinensis) cultivars reveals that glucosinolate content is correlated with carotenoid content. *Journal of Agricultural and Food Chemistry*, *64*(21), 4426–4434. doi:[10.1021/acs.jafc.6b01323](https://doi.org/10.1021/acs.jafc.6b01323)

Bell, L., Oloyede, O. O., Lignou, S., Wagstaff, C., & Methven, L. (2018). Taste and flavor perceptions of glucosinolates, isothiocyanates, and related compounds. *Molecular Nutrition and Food Research*, *62*(18), e1700990. doi:[10.1002/mnfr.201700990](https://doi.org/10.1002/mnfr.201700990), [29578640](http://www.ncbi.nlm.nih.gov/pubmed/29578640)

Bernal, J., González, D., Valverde, S., Toribio, L., & Ares, A. M. (2019). Improved separation of intact glucosinolates in bee pollen by using ultra-high-performance liquid chromatography coupled to quadrupole time-of-flight mass spectrometry. *Food Analytical Methods*, *12*(5), 1170–1178. doi:[10.1007/s12161-019-01446-2](https://doi.org/10.1007/s12161-019-01446-2)

Capriotti, A. L., Cavaliere, C., La Barbera, G., Montone, C. M., Piovesana, S., Zenezini Chiozzi, R. Z., & Laganà, A. (2018). Chromatographic column evaluation for the untargeted profiling of glucosinolates in cauliflower by means of ultra-high performance liquid chromatography coupled to high resolution mass spectrometry. *Talanta, 179*, 792–802. doi:[10.1016/j.talanta.2017.12.019](https://doi.org/10.1016/j.talanta.2017.12.019)

Chun, J. H., Kim, N. H., Seo, M. S., Jin, M., Park, S. U., Arasu, M. V., Kim, S. J., & Al-Dhabi, N. A. (2018). Molecular characterization of glucosinolates and carotenoid biosynthetic genes in Chinese cabbage (*Brassica rapa* L. ssp. *pekinensis*). *Saudi Journal of Biological Sciences*, *25*(1), 71–82. doi:[10.1016/j.sjbs.2016.04.004](https://doi.org/10.1016/j.sjbs.2016.04.004)

Ciska, E., Honke, J., & Drabińska, N. (2021). Changes in glucosinolates and their breakdown products during the fermentation of cabbage and prolonged storage of sauerkraut: Focus on sauerkraut juice. *Food Chemistry*, *365*, 130498. doi:[10.1016/j.foodchem.2021.130498](https://doi.org/10.1016/j.foodchem.2021.130498)

Codex Alimentarius Commission. (2001). Codex standard for Kimchi. CXS 223-2001. Retrieved March 25, 2022. <https://www.fao.org/fao-who-codexalimentarius/sh-proxy/es/?lnk=1&url=https%253A%252F%252Fworkspace.fao.org%252Fsites%252Fcodex%252FStandards%252FCXS%2B223-2001%252FCXS_223e.pdf>

Esteve, M. (2020). Mechanisms underlying biological effects of cruciferous glucosinolate-derived isothiocyanates/indoles: A focus on metabolic syndrome. *Frontiers in Nutrition, 7,* 111. doi:10.3389/fnut.2020.00111

Fuentes, F., Paredes-Gonzalez, X., & Kong, A. N. (2015). Dietary glucosinolates sulforaphane, phenethyl isothiocyanate, indole-3-carbinol/3, 3′-diindolylmethane: Antioxidative stress/inflammation, Nrf2, epigenetics/epigenomics and in vivo cancer chemopreventive efficacy. *Current Pharmacology Reports*, *1*(3), 179–196. doi:[10.1007/s40495-015-0017-y](https://doi.org/10.1007/s40495-015-0017-y)

Hanschen, F. S., Lamy, E., Schreiner, M., & Rohn, S. (2014). Reactivity and stability of glucosinolates and their breakdown products in foods. *Angewandte Chemie*, *53*(43), 11430–11450. doi:[10.1002/anie.201402639](https://doi.org/10.1002/anie.201402639)

Hennig, K., Verkerk, R., Bonnema, G., & Dekker, M. (2012). Pitfalls in the desulphation of glucosinolates in a high-throughput assay. *Food Chemistry*, *134*(4), 2355–2361. doi:[10.1016/j.foodchem.2012.04.015](https://doi.org/10.1016/j.foodchem.2012.04.015)

Hooshmand, K., & Fomsgaard, I. S. (2021). Analytical methods for quantification and identification of intact glucosinolates in *Arabidopsis* roots using LC-QqQ (LIT)-MS/MS. *Metabolites*, *11*(1), 47. doi:[10.3390/metabo11010047](https://doi.org/10.3390/metabo11010047)

KATI, Korea agricultural trade information. Kimchi, Import and export in the last 5 years. (2020). Retrieved March 25, 2022. <https://www.kati.net/product/basisInfo.do?lcdCode=MD161>

KFRI, Korea Food Research Institute. (2006). Development of standard index and study of labeling plan for pungency, fermentation grade of kimchi. Retrieved March 25, 2022. file:///C:/Users/user/Downloads/%EB%B3%B4%EA%B3%A0%EC%84%9C%20(14).pdf

KHIDI, Korea Health Industry Development Institute. National Health and Nutrition Examination Survey 2015. (2015). Retrieved March 25, 2022. https://www.khidi.or.kr/kps/dhraStat/result2?menuId=MENU01653&gubun=&year=2015&category=&schText=%EA%B9%80%EC%B9%98

KHIDI, Korea Health Industry Development Institute. National Health and Nutrition Examination Survey 2016. (2016). Retrieved March 25, 2022. <https://www.khidi.or.kr/kps/dhraStat/result2?menuId=MENU01653&gubun=&year=2016&category=&schText=%EA%B9%80%EC%B9%98>

KHIDI, Korea Health Industry Development Institute. National Health and Nutrition Examination Survey 2017. (2017). Retrieved March 25, 2022. https://www.khidi.or.kr/kps/dhraStat/result2?menuId=MENU01653&gubun=&year=2017&category=&schText=%EA%B9%80%EC%B9%98

KHIDI, Korea Health Industry Development Institute. National Health and Nutrition Examination Survey 2018. (2018). Retrieved March 25, 2022. <https://www.khidi.or.kr/kps/dhraStat/result2?menuId=MENU01653&gubun=&year=2018&category=&schText=%EA%B9%80%EC%B9%98>

KHIDI, Korea Health Industry Development Institute. National Health and Nutrition Examination Survey 2019. (2019). Retrieved March 25, 2022. https://www.khidi.or.kr/kps/dhraStat/result2?menuId=MENU01653&gubun=&year=2019&category=&schText=%EA%B9%80%EC%B9%98

Kim, H. J., Lee, M. J., Jeong, M. H., & Kim, J. E. (2017). Identification and quantification of glucosinolates in kimchi by liquid chromatography-electrospray tandem mass spectrometry. *International Journal of Analytical Chemistry*, *2017*, 6753481. doi:[10.1155/2017/6753481](https://doi.org/10.1155/2017/6753481)

Kim, J. K., Chu, S. M., Kim, S. J., Lee, D. J., Lee, S. Y., Lim, S. H. (2010). Variation of glucosinolates in vegetable crops of *Brassica rapa* L. ssp. *pekinensis*. *Food Chemistry*, *119*(1), 423–428. doi:[10.1016/j.foodchem.2009.08.051](https://doi.org/10.1016/j.foodchem.2009.08.051)

Klopsch, R., Witzel, K., Börner, A., Schreiner, M., & Hanschen, F. S. (2017). Metabolic profiling of glucosinolates and their hydrolysis products in a germplasm collection of Brassica rapa turnips. *Food Research International*, *100*(3), 392–403. doi:[10.1016/j.foodres.2017.04.016](https://doi.org/10.1016/j.foodres.2017.04.016)

Kronbak, R., Duus, F., & Vang, O. (2010). Effect of 4-methoxyindole-3-carbinol on the proliferation of colon cancer cells in vitro, when treated alone or in combination with indole-3-carbinol. *Journal of Agricultural and Food Chemistry*, *58*(14), 8453–8459. doi:[10.1021/jf101806t](https://doi.org/10.1021/jf101806t)

Lee, M.-K., Chun, J., Byeon, D. H., Chung, S., Park, S. U., Park, S, Arasu, M.V., Al-Dhabi N.A., Lim, Y., & Kim, S. (2014). Variation of glucosinolates in 62 varieties of Chinese cabbage (*Brassica rapa* L. ssp. pekinensis) and their antioxidant activity. *LWT – Food Science and Technology*, *58*(1), 93–101. doi:[10.1016/j.lwt.2014.03.001](https://doi.org/10.1016/j.lwt.2014.03.001)

Maldini, M., Foddai, M., Natella, F., Petretto, G. L., Rourke, J. P., Chessa, M., & Pintore, G. (2017). Identification and quantification of glucosinolates in different tissues of *Raphanus raphanistrum* by liquid chromatography tandem-mass spectrometry. *Journal of Food Composition and Analysis*, *61*, 20–27. doi:[10.1016/j.jfca.2016.06.002](https://doi.org/10.1016/j.jfca.2016.06.002)

Matuszewski, B. K., Constanzer, M. L., & Chavez-Eng, C. M. (2003). Strategies for the assessment of matrix effect in quantitative bioanalytical methods based on HPLC− MS/MS. *Analytical Chemistry*, *75*(13), 3019–3030. doi:[10.1021/ac020361s](https://doi.org/10.1021/ac020361s)

Mullaney, J. A., Kelly, W. J., McGhie, T. K., Ansell, J., & Heyes, J. A. (2013). Lactic acid bacteria convert glucosinolates to nitriles efficiently yet differently from Enterobacteriaceae. *Journal of Agricultural and Food Chemistry*, *61*(12), 3039–3046. doi:[10.1021/jf305442j](https://doi.org/10.1021/jf305442j)

Padilla, G., Cartea, M. E., Velasco, P., de Haro, A., & Ordás, A. (2007). Variation of glucosinolates in vegetable crops of *Brassica rapa*. *Phytochemistry*, *68*(4), 536–545. doi:[10.1016/j.phytochem.2006.11.017](https://doi.org/10.1016/j.phytochem.2006.11.017)

Shakour, Z. T., Shehab, N. G., Gomaa, A. S., Wessjohann, L. A., & Farag, M. A. (2022). Metabolic and biotransformation effects on dietary glucosinolates, their bioavailability, catabolism and biological effects in different organisms. *Biotechnology Advances*, 54, 107784. doi:[10.1016/j.biotechadv.2021.107784](https://doi.org/10.1016/j.biotechadv.2021.107784)

Thomas, M., Badr, A., Desjardins, Y., Gosselin, A., & Angers, P. (2018). Characterization of industrial broccoli discards (*Brassica oleracea* var. italica) for their glucosinolate, polyphenol and flavonoid contents using UPLC MS/MS and spectrophotometric methods. *Food Chemistry*, *245*, 1204–1211. doi:[10.1016/j.foodchem.2017.11.021](https://doi.org/10.1016/j.foodchem.2017.11.021)

Tolonen, M., Taipale, M., Viander, B., Pihlava, J. M., Korhonen, H., & Ryhänen, E. L. (2002). Plant-derived biomolecules in fermented cabbage. *Journal of Agricultural and Food Chemistry*, *50*(23), 6798–6803. doi:[10.1021/jf0109017](https://doi.org/10.1021/jf0109017)

United States Food and Drug Administration. (2019). Guidelines for the validation of chemical methods for the FDA FVM program. Retrieved March 25, 2022. <https://www.fda.gov/media/81810/download>

Zhou, W., Yang, S., & Wang, P. G. (2017). Matrix effects and application of matrix effect factor. *Bioanalysis*, *9*(23), 1839–1844. doi:[10.4155/bio-2017-0214](https://doi.org/10.4155/bio-2017-0214)

**Figure legend**

**Figure 1.** General proportions of glucosinolates in kimchi samples in different fermentation stages. GAL, glucoalyssin; GBN, glucobrassicanapin; GBS, glucobrassicin; GBT, glucoberteroin; GCH, glucocheirolin; GIB, glucoiberin; GNA, gluconapin; GNS, gluconasturtiin; GRA, glucoraphanin; GRE, glucoraphenin; NEO, neoglucobrassicin PRO, progoitrin; SIN, sinigrin; 4ME, 4-methoxyglucobrassicin

**Table 1.** Matrix effect evaluation results for intact glucosinolates

| Glucosinolate | Matrix effect (%) | | |
| --- | --- | --- | --- |
|  | Low | Medium | High |
| Glucoiberin | 102 ± 7 | 103 ± 3 | 103 ± 2 |
| Glucocheirolin | 102 ± 2 | 103 ± 3 | 102 ± 2 |
| Progoitrin | 104 ± 5 | 101 ± 3 | 103 ± 1 |
| Sinigrin | 101 ± 4 | 102 ± 3 | 102 ± 2 |
| Glucoraphanin | 98 ± 4 | 100 ± 2 | 103 ± 2 |
| Glucoraphenin | 100 ± 4 | 104 ± 4 | 105 ± 2 |
| Glucoalyssin | 100 ± 8 | 102 ± 2 | 103 ± 2 |
| Gluconapin | 99 ± 4 | 102 ± 2 | 101 ± 1 |
| Glucobrassicanapin | 102 ± 7 | 105 ± 5 | 102 ± 2 |
| Glucobrassicin | 105 ± 7 | 100 ± 3 | 103 ± 3 |
| Glucoberteroin | 104 ± 5 | 100 ± 1 | 101 ± 1 |
| Gluconasturtiin | 105 ± 2 | 102 ± 3 | 102 ± 1 |
| 4-Methoxyglucobrassicin | 88 ± 4 | 90 ± 4 | 91 ± 5 |
| Neoglucobrassicin | 100 ± 1 | 100 ± 1 | 102 ± 1 |

**Table 2.** Linear ranges, coefficients of determination (r^2^), and limits of detection (LOD) and quantification (LOQ) for intact glucosinolates

| Glucosinolate | Linear range (nmol/L) | r^2^ | LOD (nmol/L) | LOQ (nmol/L) |
| --- | --- | --- | --- | --- |
| Glucoiberin | 15–1000 | 0.9997 | 5 | 15 |
| Glucocheirolin | 16–1000 | 0.9998 | 5 | 16 |
| Progoitrin | 21–1000 | 0.9997 | 7 | 21 |
| Sinigrin | 27–1000 | 0.9991 | 9 | 27 |
| Glucoraphanin | 19–1000 | 0.9993 | 6 | 19 |
| Glucoraphenin | 21–1000 | 0.9997 | 7 | 21 |
| Glucoalyssin | 5–1000 | 0.9998 | 2 | 5 |
| Gluconapin | 14–1000 | 0.9995 | 5 | 14 |
| Glucobrassicanapin | 10–1000 | 0.9995 | 3 | 10 |
| Glucobrassicin | 21–1000 | 0.9996 | 7 | 21 |
| Glucoberteroin | 15–1000 | 0.9998 | 5 | 15 |
| Gluconasturtiin | 16–1000 | 0.9997 | 5 | 16 |
| 4-Methoxyglucobrassicin | 35–3000 | 0.9994 | 11 | 35 |
| Neoglucobrassicin | 12–1000 | 0.9998 | 4 | 12 |

**Table 3.** Accuracy evaluation results for intact glucosinolates

| Glucosinolate | Recovery (%) | | |
| --- | --- | --- | --- |
|  | Low | Medium | High |
| Glucoiberin | 90 ± 5 | 88 ± 4 | 94 ± 4 |
| Glucocheirolin | 89 ± 5 | 90 ± 5 | 96 ± 5 |
| Progoitrin | 84 ± 5 | 89 ± 4 | 91 ± 4 |
| Sinigrin | 91 ± 5 | 91 ± 7 | 92 ± 4 |
| Glucoraphanin | 92 ± 7 | 93 ± 3 | 94 ± 7 |
| Glucoraphenin | 90 ± 7 | 92 ± 6 | 93 ± 4 |
| Glucoalyssin | 89 ± 6 | 95 ± 7 | 101 ± 4 |
| Gluconapin | 89 ± 6 | 87 ± 4 | 94 ± 4 |
| Glucobrassicanapin | 92 ± 6 | 91 ± 7 | 96 ± 6 |
| Glucobrassicin | 89 ± 6 | 91 ± 3 | 95 ± 4 |
| Glucoberteroin | 83 ± 6 | 82 ± 4 | 86 ± 3 |
| Gluconasturtiin | 87 ± 3 | 88 ± 4 | 92 ± 2 |
| 4-Methoxyglucobrassicin | 85 ± 6 | 86 ± 4 | 89 ± 7 |
| Neoglucobrassicin | 88 ± 4 | 90 ± 3 | 94 ± 3 |

**Table 4.** Intra- and interday precision evaluation results for intact glucosinolates

| Glucosinolate | Intraday relative standard deviation (%) | | | Interday relative standard deviation (%) | | |
| --- | --- | --- | --- | --- | --- | --- |
|  | Low | Medium | High | Low | Medium | High |
| Glucoiberin | 5 | 5 | 5 | 6 | 6 | 4 |
| Glucocheirolin | 5 | 6 | 5 | 5 | 6 | 4 |
| Progoitrin | 6 | 4 | 4 | 7 | 5 | 4 |
| Sinigrin | 6 | 8 | 4 | 7 | 7 | 4 |
| Glucoraphanin | 8 | 3 | 7 | 8 | 6 | 5 |
| Glucoraphenin | 8 | 7 | 5 | 7 | 6 | 5 |
| Glucoalyssin | 7 | 7 | 4 | 8 | 8 | 4 |
| Gluconapin | 6 | 5 | 4 | 7 | 6 | 3 |
| Glucobrassicanapin | 7 | 8 | 6 | 8 | 8 | 6 |
| Glucobrassicin | 7 | 3 | 5 | 7 | 6 | 4 |
| Glucoberteroin | 8 | 5 | 4 | 7 | 4 | 3 |
| Gluconasturtiin | 3 | 5 | 2 | 6 | 6 | 3 |
| 4-Methoxyglucobrassicin | 7 | 5 | 7 | 7 | 5 | 7 |
| Neoglucobrassicin | 4 | 3 | 3 | 5 | 5 | 2 |

**Table 5.** Glucosinolate contents (nmol/g dry weight) of kimchi samples with different fermentation grade

| Sample^1)^ | | Glucosinolates^2)^ | | | | | | | | | | | | | | |
| --- | --- | --- | --- | --- | --- | --- | --- | --- | --- | --- | --- | --- | --- | --- | --- | --- |
|  |  | GIB | GCH | PRO | SIN | GRA | GRE | GAL | GNA | GBN | GBS | GBT | GNS | 4ME | NEO | Total |
| K1 | N | ND^3)^ | ND | 48 ± 2 | ND | <LOD^4)^ | 23 ± 1 | 78 ± 1 | 100 ± 1 | 280 ± 4 | 7 ± 0 | 39 ± 1 | 19 ± 0 | 556 ± 22 | 23 ± 0 | 1172 ± 25 |
|  | M | ND | ND | <LOD | ND | <LOQ^5)^ | ND | 12 ± 1 | <LOD | 3 ± 0 | <LOQ | 3 ± 0 | <LOQ | 332 ± 1 | ND | 351 ± 2 |
|  | O | ND | ND | <LOD | ND | ND | ND | 2 ± 0 | <LOD | 2 ± 0 | ND | <LOD | <LOD | 28 ± 1 | <LOD | 32 ± 1 |
| K2 | N | ND | ND | 15 ± 1 | ND | <LOQ | <LOQ | 32 ± 1 | 52 ± 1 | 121 ± 1 | 18 ± 1 | 14 ± 0 | 7 ± 0 | 748 ± 24 | 30 ± 0 | 1037 ± 26 |
|  | M | ND | ND | 13 ± 0 | ND | <LOD | ND | 24 ± 0 | 21 ± 1 | 69 ± 1 | 11 ± 0 | 11 ± 0 | 7 ± 0 | 430 ± 9 | 16 ± 0 | 601 ± 10 |
|  | O | ND | ND | ND | ND | ND | ND | ND | ND | <LOD | ND | ND | ND | <LOQ | ND | 0 ± 0 |
| K3 | N | ND | ND | 7 ± 1 | ND | <LOQ | <LOD | 28 ± 0 | 17 ± 0 | 37 ± 1 | <LOQ | 11 ± 0 | 3 ± 0 | 334 ± 5 | 5 ± 0 | 443 ± 5 |
|  | M | ND | ND | 4 ± 0 | ND | <LOD | ND | 10 ± 0 | 9 ± 1 | 19 ± 1 | <LOD | 4 ± 0 | <LOQ | 118 ± 2 | <LOQ | 164 ± 3 |
|  | O | ND | ND | ND | ND | ND | ND | 1 ± 0 | <LOD | <LOQ | ND | ND | ND | 39 ± 0 | ND | 41 ± 1 |
| K4 | N | ND | ND | 40 ± 1 | <LOD | 5 ± 0 | <LOD | 62 ± 2 | 139 ± 2 | 191 ± 1 | 41 ± 2 | 40 ± 1 | 15 ± 1 | 768 ± 7 | 24 ± 0 | 1324 ± 12 |
|  | M | ND | ND | 21 ± 1 | <LOD | <LOQ | ND | 29 ± 1 | 96 ± 3 | 100 ± 2 | 15 ± 0 | 9 ± 1 | 7 ± 0 | 368 ± 10 | 17 ± 0 | 664 ± 6 |
|  | O | ND | ND | ND | ND | ND | ND | 1 ± 0 | <LOD | <LOQ | ND | ND | ND | 56 ± 1 | ND | 57 ± 1 |
| K5 | N | ND | ND | 11 ± 1 | ND | 4 ± 0 | 8 ± 1 | 78 ± 1 | 43 ± 1 | 77 ± 1 | 8 ± 0 | 33 ± 0 | 11 ± 0 | 708 ± 14 | 24 ± 0 | 1005 ± 15 |
|  | M | ND | ND | <LOQ | ND | ND | ND | 8 ± 0 | 8 ± 0 | 22 ± 0 | <LOD | 4 ± 0 | <LOQ | 197 ± 4 | 2 ± 0 | 241 ± 5 |
|  | O | ND | ND | ND | ND | ND | ND | 1 ± 0 | <LOD | <LOQ | ND | ND | ND | 56 ± 1 | ND | 57 ± 1 |
| K6 | N | ND | ND | 50 ± 0 | ND | <LOQ | 15 ± 0 | 63 ± 2 | 73 ± 1 | 169 ± 3 | 45 ± 2 | 48 ± 1 | 16 ± 1 | 776 ± 19 | 32 ± 1 | 1286 ± 17 |
|  | M | ND | ND | 14 ± 1 | ND | <LOD | ND | 25 ± 0 | 29 ± 1 | 84 ± 1 | 23 ± 0 | 14 ± 1 | 6 ± 0 | 262 ± 10 | 10 ± 0 | 466 ± 12 |
|  | O | ND | ND | <LOD | ND | ND | ND | 2 ± 0 | <LOQ | 4 ± 0 | <LOD | <LOD | <LOD | <LOD | <LOD | 5 ± 0 |
| K7 | N | ND | ND | 51 ± 2 | ND | <LOQ | 30 ± 1 | 48 ± 0 | 110 ± 2 | 290 ± 7 | 14 ± 1 | 20 ± 0 | 22 ± 1 | 557 ± 9 | 69 ± 2 | 1212 ± 16 |
|  | M | ND | ND | 33 ± 1 | ND | <LOQ | <LOD | 37 ± 1 | 100 ± 0 | 212 ± 4 | 14 ± 1 | 14 ± 0 | 21 ± 0 | 349 ± 5 | 11 ± 0 | 792 ± 5 |
|  | O | ND | ND | <LOD | ND | ND | ND | 2 ± 0 | <LOQ | 3 ± 0 | <LOD | <LOD | <LOD | <LOQ | <LOD | 5 ± 0 |
| K8 | N | ND | ND | 20 ± 1 | <LOD | 8 ± 1 | 31 ± 1 | 129 ± 2 | 191 ± 0 | 305 ± 4 | 135 ± 2 | 25 ± 1 | 28 ± 0 | 966 ± 7 | 62 ± 1 | 1902 ± 11 |
|  | M | ND | ND | 16 ± 0 | ND | 5 ± 0 | ND | 101 ± 1 | 147 ± 1 | 291 ± 3 | 48 ± 1 | 22 ± 0 | 28 ± 1 | 632 ± 15 | 31 ± 1 | 1320 ± 10 |
|  | O | ND | ND | ND | ND | ND | ND | 1 ± 0 | <LOD | <LOQ | <LOD | <LOD | <LOD | 37 ± 1 | <LOD | 38 ± 1 |
| K9 | N | ND | ND | 56 ± 1 | <LOD | <LOQ | 21 ± 0 | 113 ± 2 | 349 ± 7 | 938 ± 2 | 123 ± 1 | 35 ± 0 | 40 ± 1 | 797 ± 12 | 183 ± 1 | 2655 ± 14 |
|  | M | ND | ND | 34 ± 1 | <LOD | <LOQ | <LOD | 94 ± 2 | 202 ± 3 | 475 ± 0 | 38 ± 1 | 28 ± 1 | 25 ± 0 | 653 ± 9 | 52 ± 0 | 1602 ± 7 |
|  | O | ND | ND | <LOD | ND | ND | ND | 2 ± 0 | <LOQ | 4 ± 0 | <LOD | <LOD | <LOD | 79 ± 1 | <LOD | 85 ± 1 |
| K10 | N | ND | ND | 77 ± 1 | ND | <LOQ | ND | 100 ± 1 | 169 ± 2 | 356 ± 7 | 216 ± 3 | 16 ± 0 | 35 ± 1 | 1483 ± 36 | 1003 ± 10 | 3458 ± 23 |
|  | M | ND | ND | 54 ± 2 | ND | <LOQ | ND | 25 ± 1 | 25 ± 0 | 164 ± 1 | 58 ± 1 | 10 ± 0 | 24 ± 0 | 1344 ± 20 | 101 ± 2 | 1806 ± 24 |
|  | O | ND | ND | <LOQ | ND | ND | ND | 3 ± 0 | 3 ± 0 | 8 ± 0 | 9 ± 1 | <LOD | <LOQ | 242 ± 6 | 40 ± 1 | 306 ± 8 |
| K11 | N | ND | ND | 102 ± 4 | ND | 6 ± 0 | 6 ± 0 | 50 ± 3 | 5 ± 0 | 57 ± 2 | 120 ± 7 | 23 ± 0 | 26 ± 1 | 679 ± 9 | 230 ± 2 | 1305 ± 11 |
|  | M | ND | ND | 40 ± 1 | ND | <LOQ | ND | 31 ± 1 | 5 ± 0 | 47 ± 1 | 26 ± 1 | 18 ± 1 | 20 ± 0 | 253 ± 2 | 62 ± 1 | 502 ± 1 |
|  | O | ND | ND | ND | ND | ND | ND | 1 ± 0 | ND | <LOD | ND | ND | <LOD | 26 ± 2 | <LOD | 27 ± 2 |
| K12 | N | ND | ND | 126 ± 2 | <LOQ | 9 ± 1 | 6 ± 0 | 345 ± 12 | 906 ± 6 | 1318 ± 3 | 40 ± 0 | 153 ± 1 | 93 ± 0 | 562 ± 4 | 48 ± 0 | 3606 ± 15 |
|  | M | ND | ND | 94 ± 1 | <LOD | <LOQ | <LOD | 187 ± 0 | 412 ± 8 | 702 ± 8 | 19 ± 1 | 105 ± 1 | 49 ± 0 | 394 ± 6 | 26 ± 0 | 1988 ± 8 |
|  | O | ND | ND | <LOD | ND | ND | ND | 2 ± 0 | 3 ± 0 | 6 ± 0 | <LOD | <LOD | <LOD | 11 ± 1 | <LOD | 22 ± 0 |
| K13 | N | ND | ND | 53 ± 1 | <LOD | 4 ± 1 | 31 ± 1 | 190 ± 2 | 403 ± 15 | 908 ± 11 | 52 ± 2 | 72 ± 0 | 35 ± 0 | 560 ± 11 | 46 ± 1 | 2354 ± 19 |
|  | M | ND | ND | 14 ± 0 | <LOD | <LOQ | <LOD | 71 ± 1 | 218 ± 1 | 443 ± 6 | 11 ± 1 | 36 ± 1 | 24 ± 0 | 264 ± 6 | 21 ± 0 | 1101 ± 6 |
|  | O | ND | ND | ND | ND | ND | ND | 2 ± 0 | 4 ± 0 | 7 ± 0 | ND | <LOD | <LOD | 22 ± 1 | ND | 35 ± 1 |
| K14 | N | ND | ND | 81 ± 2 | ND | 4 ± 0 | <LOQ | 241 ± 12 | 328 ± 3 | 795 ± 17 | 130 ± 4 | 45 ± 1 | 63 ± 1 | 527 ± 2 | 99 ± 0 | 2314 ± 30 |
|  | M | ND | ND | 7 ± 0 | ND | <LOQ | ND | 29 ± 1 | 147 ± 3 | 154 ± 1 | 56 ± 2 | 7 ± 0 | 16 ± 0 | 200 ± 5 | 84 ± 1 | 701 ± 11 |
|  | O | ND | ND | <LOD | ND | ND | ND | 1 ± 0 | <LOQ | 3 ± 0 | ND | <LOD | <LOD | 7 ± 0 | <LOD | 11 ± 0 |
| K15 | N | ND | ND | 24 ± 0 | ND | 5 ± 0 | <LOQ | 55 ± 1 | 57 ± 1 | 92 ± 1 | 25 ± 1 | 27 ± 0 | 12 ± 0 | 813 ± 11 | 123 ± 1 | 1233 ± 10 |
|  | M | ND | ND | 15 ± 1 | ND | <LOQ | ND | 17 ± 1 | 9 ± 0 | 26 ± 0 | 4 ± 0 | 4 ± 0 | 8 ± 0 | 223 ± 7 | 4 ± 0 | 310 ± 7 |
|  | O | ND | ND | <LOQ | ND | ND | ND | 8 ± 1 | 7 ± 0 | 15 ± 0 | <LOQ | <LOQ | <LOQ | 62 ± 3 | <LOQ | 92 ± 3 |
| K16 | N | ND | ND | 213 ± 6 | ND | 6 ± 0 | 6 ± 0 | 82 ± 2 | 28 ± 1 | 466 ± 5 | 73 ± 1 | 114 ± 1 | 71 ± 0 | 672 ± 35 | 290 ± 2 | 2019 ± 46 |
|  | M | ND | ND | 54 ± 1 | ND | <LOD | ND | 19 ± 1 | 11 ± 0 | 163 ± 2 | 24 ± 1 | 9 ± 0 | 29 ± 0 | 220 ± 5 | 38 ± 0 | 567 ± 7 |
|  | O | ND | ND | <LOD | ND | ND | ND | 2 ± 0 | <LOD | 4 ± 0 | <LOD | ND | <LOD | 38 ± 0 | <LOQ | 43 ± 1 |
| K17 | N | ND | ND | 37 ± 1 | ND | 4 ± 0 | 5 ± 0 | 56 ± 1 | 100 ± 2 | 268 ± 2 | 57 ± 2 | 32 ± 0 | 54 ± 0 | 421 ± 11 | 26 ± 0 | 1061 ± 9 |
|  | M | ND | ND | <LOQ | ND | ND | ND | 5 ± 0 | 7 ± 0 | 25 ± 1 | 12 ± 0 | <LOQ | 8 ± 0 | 138 ± 1 | 25 ± 0 | 218 ± 1 |
|  | O | ND | ND | ND | ND | ND | ND | ND | ND | ND | ND | ND | ND | <LOD | ND | 0 ± 0 |
| K18 | N | ND | ND | 95 ± 2 | ND | 8 ± 1 | ND | 119 ± 3 | 29 ± 1 | 318 ± 1 | 57 ± 2 | 45 ± 1 | 61 ± 1 | 230 ± 5 | 22 ± 0 | 985 ± 8 |
|  | M | ND | ND | <LOQ | ND | ND | ND | 4 ± 0 | <LOD | 10 ± 0 | <LOD | <LOQ | <LOQ | 29 ± 1 | <LOQ | 43 ± 0 |
|  | O | ND | ND | ND | ND | ND | ND | 1 ± 0 | ND | <LOQ | ND | ND | <LOD | <LOD | ND | 1 ± 0 |
| K19 | N | ND | ND | 123 ± 2 | ND | 5 ± 1 | 14 ± 1 | 74 ± 2 | 36 ± 0 | 295 ± 3 | 47 ± 1 | 53 ± 1 | 53 ± 1 | 219 ± 3 | 16 ± 0 | 935 ± 5 |
|  | M | ND | ND | <LOD | ND | ND | ND | 1 ± 0 | ND | 5 ± 0 | <LOD | ND | <LOQ | 21 ± 0 | <LOQ | 27 ± 1 |
|  | O | ND | ND | ND | ND | ND | ND | ND | ND | <LOD | ND | ND | ND | <LOD | ND | 0 ± 0 |
| K20 | N | ND | ND | 21 ± 0 | <LOD | 11 ± 0 | 81 ± 1 | 125 ± 3 | 474 ± 14 | 883 ± 21 | 178 ± 1 | 46 ± 1 | 126 ± 1 | 580 ± 4 | 96 ± 2 | 2620 ± 37 |
|  | M | ND | ND | 14 ± 0 | ND | ND | ND | 16 ± 0 | 76 ± 1 | 202 ± 5 | 12 ± 1 | 3 ± 0 | 26 ± 1 | 172 ± 4 | 11 ± 0 | 532 ± 9 |
|  | O | ND | ND | ND | ND | ND | ND | 1 ± 0 | <LOD | <LOQ | <LOD | ND | <LOD | <LOQ | <LOD | 1 ± 0 |

^1)^N, non-fermented; M, moderate-fermented; and O, over-fermented

^2)^GIB, glucoiberin; GCH, glucocheirolin; PRO, progoitrin; SIN, sinigrin; GRA, glucoraphanin; GRE, glucoraphenin; GAL, glucoalyssin; GNA, gluconapin; GBN, glucobrassicanapin; GBS, glucobrassicin; GBT, glucoberteroin; GNS, gluconasturtiin; 4ME, 4-methoxyglucobrassicin; NEO, neoglucobrassicin

^3)^ND, not detected

^4)^LOD, limit of detection

^5)^LOQ, limit of quantification
